# Evaluating Alzheimer’s Disease Progression Using Rate of Regional Hippocampal Atrophy

**DOI:** 10.1371/journal.pone.0071354

**Published:** 2013-08-12

**Authors:** Edit Frankó, Olivier Joly, for the Alzheimer’s Disease Neuroimaging Initiative

**Affiliations:** 1 INSERM U1075, Université de Caen Basse-Normandie, Caen, France; 2 Department of Neurodegenerative Disease, Institute of Neurology, University College London, London, United Kingdom; 3 National Prion Clinic, Institute of Neurology, University College London, London, United Kingdom; 4 NeuroSpin, Gif-sur-Yvette, France; 5 Institute of Neuroscience, Newcastle University, Newcastle upon Tyne, United Kingdom; University of Manchester, United Kingdom

## Abstract

Alzheimer’s disease (AD) is characterized by neurofibrillary tangle and neuropil thread deposition, which ultimately results in neuronal loss. A large number of magnetic resonance imaging studies have reported a smaller hippocampus in AD patients as compared to healthy elderlies. Even though this difference is often interpreted as atrophy, it is only an indirect measurement. A more direct way of measuring the atrophy is to use repeated MRIs within the same individual. Even though several groups have used this appropriate approach, the pattern of hippocampal atrophy still remains unclear and difficult to relate to underlying pathophysiology. Here, in this longitudinal study, we aimed to map hippocampal atrophy rates in patients with AD, mild cognitive impairment (MCI) and elderly controls. Data consisted of two MRI scans for each subject. The symmetric deformation field between the first and the second MRI was computed and mapped onto the three-dimensional hippocampal surface. The pattern of atrophy rate was similar in all three groups, but the rate was significantly higher in patients with AD than in control subjects. We also found higher atrophy rates in progressive MCI patients as compared to stable MCI, particularly in the antero-lateral portion of the right hippocampus. Importantly, the regions showing the highest atrophy rate correspond to those that were described to have the highest burden of tau deposition. Our results show that local hippocampal atrophy rate is a reliable biomarker of disease stage and progression and could also be considered as a method to objectively evaluate treatment effects.

## Introduction

Alzheimer’s disease (AD) is the most common form of dementia in the elderly population [1]. In 2010, the estimated number of patients with AD worldwide was 35.6 million, and this number is predicted to triple by 2050 (World Alzheimer Report, 2010). These numbers highlight a pressing need to develop disease-modifying treatments. Existing treatments are only effective in the early phase of the disease, and even then, their effect is highly variable among patients [2].

At present, the clinical diagnosis of AD requires that the patient has dementia [3], which is already associated with the widespread deposition of amyloid plaques and neurofibrillary tangles in the brain [4]. Indeed, amyloid and tau deposition in the entorhinal cortex has been detected in clinically silent cases [5]. Even in mild AD, these neuropathological changes cause neuronal loss in the entorhinal cortex and hippocampus [6], and these changes result in decreased volume [7]. Many magnetic resonance imaging (MRI) studies have indeed reported smaller volumes in AD patients than in controls [8]–[10], which indirectly reflects faster atrophy in AD. However, a more direct way of measuring the atrophy is the use of repeated MRI scans within the same individual. This can then be used to monitor the disease progression in patients. More and more volumetric studies used longitudinal dataset and reported a higher rate of hippocampal volume loss in patients with AD than in elderly controls [11]–[17] However, global hippocampal volumetry is not always sensitive enough to follow changes within a single population [18], which may reflect conversion from healthy state or disease progression. Therefore, recent studies focus on changes in hippocampal shape. Examining the shape of the hippocampus gives not only more sensitivity to follow the progression of the atrophy, but also allows the evaluation of atrophy in the different parts of the hippocampus. Thompson et al. [19] and Morra et al. [20], [21] reported that atrophy indeed varies by hippocampal sub-region. Based on differences in atrophy, they could distinguish healthy subjects from patients with AD and demonstrated correlation between atrophy and cognitive decline. However, their atrophy maps differed notably; Thompson et al. [19] described higher atrophy in the supero-lateral side of the hippocampus in AD compared to controls, whereas Morra et al. [20] found the main difference between groups in the inferior hippocampal surface. Surprisingly, the regions showing significant difference between groups did not correspond to the regions with significant atrophy in AD. These contradictory results ask for further research to find more precise methods to measure the hippocampal deformation during the progression of the disease.

In the present longitudinal study, we examined the hippocampal atrophy rate and compared its topography among healthy people and patients with MCI and AD to identify areas that can distinguish between groups. We found similar patterns of atrophy among the groups. Regional higher atrophy rate was found in AD as compared to controls. These regions also distinguish patients with stable MCI from those who eventually progressed to AD. Finally, the atrophy pattern is in agreement with the known regional anatomic specificity of tau deposition.

## Methods

### Subjects

Data used in the preparation of this article were obtained from the Alzheimer’s Disease Neuroimaging Initiative (ADNI) database (adni.loni.ucla.edu), for up-to-date information, see www.adni-info.org. The data were analysed anonymously, using publicly available secondary data from the ADNI study, therefore no ethics statement is required for this work.

From the ADNI database, a preliminary dataset containing AD and normal subjects were downloaded first while developing the method [22]. From this first dataset, we selected the subjects who had at least 2 scans with at least 200 days apart. This resulted in 90 AD patients and 54 control subjects. Later we expanded the dataset to have similar number of controls as patients and added the MCI group. From the downloaded subjects, we included the first about 90 subjects who fulfilled the inclusion criteria, namely having at least two MRIs with at least 200 days apart. Few control subjects, who turned to MCI or AD during the follow-up period, were then removed from the statistical tests. The final number of subjects included in this study were 85 healthy controls, 102 patients diagnosed with MCI and 90 patients with AD. An overview of the subject groups is provided in Table 1, including the total number of subjects, their age, sex, mini-mental state examination (MMSE) and clinical dementia rating (CDR) scores. The interscan intervals are also shown for each group. We further divided the MCI group into progressive MCI (pMCI, n = 39), containing subjects who converted to AD between the baseline and the last scan (CDR>0.5), and stable MCI (sMCI, n = 44), containing subjects with CDR scores of 0.5 at the time of the last scan. The subjects, whose CDR score was not available at the time of the last scan, were not included into this analysis. Almost all patients with AD (88 out of 90) received medication (cholinesterase inhibitors and NMDA-receptor antagonists), as did 78% of the patients with MCI (80 out of 102) whereas none of the controls.

**Table 1:** Characteristics of subjects in each group.

Group	Age in years	MMSE	CDR	Interscan time in days
Control (n = 85) F:37 M:48	76 (5)	29.2 (1)	0 (0)	1217 (303)
MCI (n = 102) F:34 M:68	75 (7)	26.8 (1.8)	0.5 (0)	1030 (393)
AD (n = 90) F:42 M:48	75 (7)	23.3 (2.2)	0.83 (0.35)	652 (188)

Mean values are followed by standard deviations (SD). MMSE: mini-mental state examination; CDR: clinical dementia rating; F: female; M:male.

### MRI Acquisitions

Structural brain MRIs were acquired at multiple ADNI sites using 1.5 Tesla MRI scanners manufactured by General Electric Healthcare, Siemens Medical Solutions, and Philips Medical Systems. Sagittal 3D MP-RAGE sequences were acquired using standard ADNI protocols (adni.loni.ucla.edu). MRI acquisition parameters were as follows: repetition time 8–9 ms, echo time 3.9 ms, flip angle 8, and slice thickness 1.2 mm. In-plane resolution differed slightly among subjects: 0.94×0.94 mm, 1.25×1.25 mm, or 1.3×1.3 mm.In this study, we used two scans from each subject. MRI_acq1 was the baseline scan (first scan of the subject), and MRI_acq2 was the last available scan (through December 2011). All subjects included in the analyses had a duration of at least 200 days between the first and last scans.

### Hippocampal Segmentation

**Figure 1:**
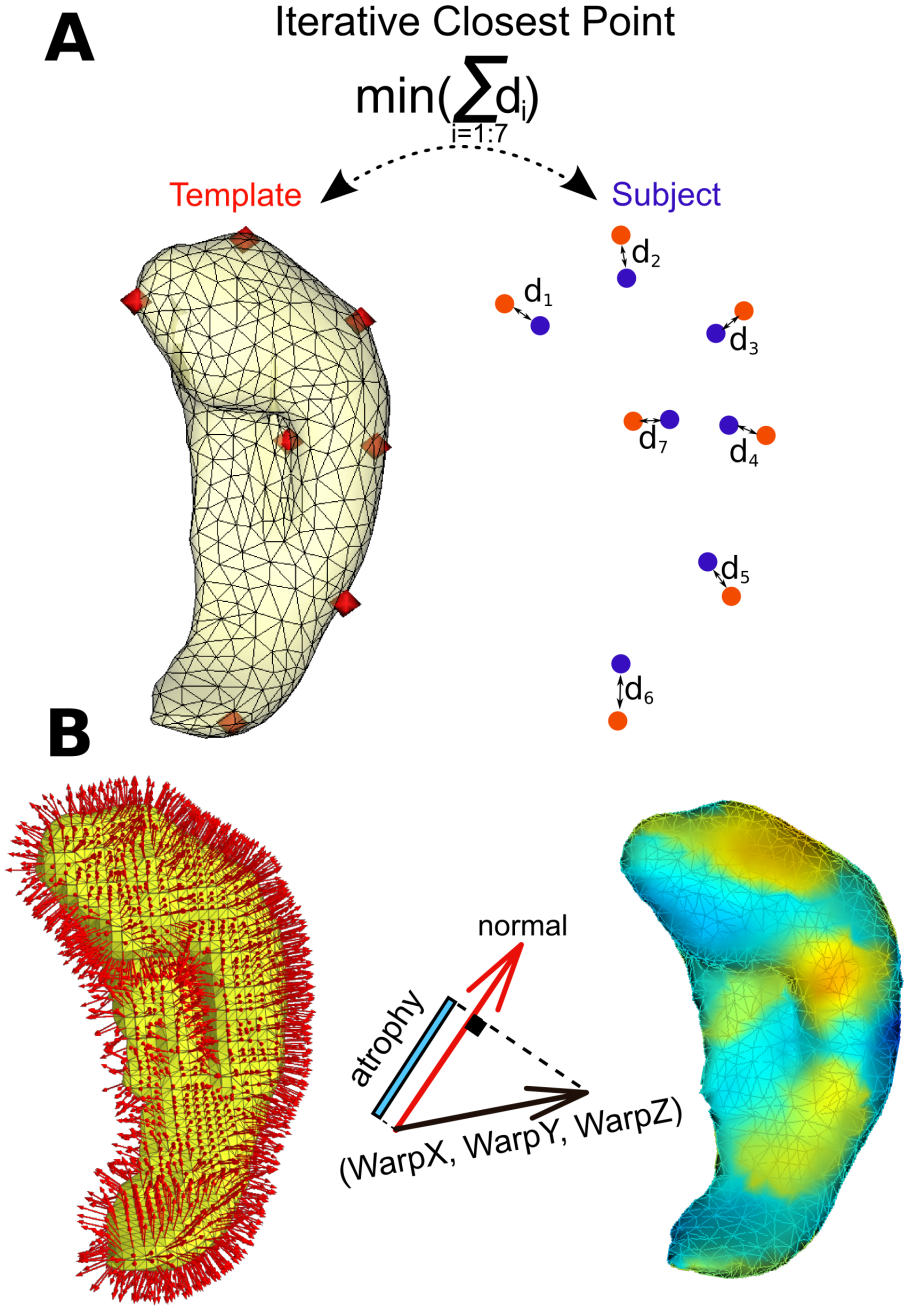
Illustration of the methods. (**A**) On the left side of this panel, we illustrate the triangulated surface of the template hippocampus and the manually defined seven points (in red). On the right side, the seven points drawn on the subject’s hippocampus are shown in blue, and the ICP algorithm is illustrated which minimises the sum of distances (di) between the red and blue points. (**B**) Illustration of the deformation field mapping onto the triangulated hippocampal surface resulting in different colours for inward (blue) and outward (orange) deformation.

A template hippocampus (right hemisphere) was manually segmented from the single-subject MNI-T1 template available in SPM (www.fil.ion.ucl.ac.uk/spm). The segmentation followed the description of Franko et al. [23] and Insausti and Amaral [24] and contains the hippocampus proper (cornu ammonis (CA) 1–3 fields), the dentate gyrus and the subiculum. For guided extraction of the subject’s hippocampus in MRI_acq1, two sets of seven points were defined in the template and in the MRI_acq1, respectively. The locations of the points are similar to those used by Pluta et al. [25]; three points were placed on coronal slices and four points on sagittal slices. The first point on the coronal view marks the appearance of the hippocampus inferior to the amygdala, the second marks the most medial point of the hippocampus at the level of the hippocampal-amygdaloid transitional area, and the third point indicates the most posterior part of the hippocampus [23]. On the sagittal view, we placed the first point on the most lateral slice of the hippocampus, the second and the third were placed 2–3 mm more medial on the anterior and posterior borders of hippocampus, respectively, and the last point was placed at the end of the intralimbic gyrus lateral to the hippocampal fissure. Finally, to extract the 3D hippocampus from MRI_acq1, the Iterative Closest Point (ICP) algorithm (www.vtk.org) was used to compute the affine transformation between the template and the subject’s hippocampus (Fig. 1A).

### Measurements of Atrophy

Following the recommendation of Yushkevich et al. [26], we performed an unbiased symmetric deformation field estimation to measure the hippocampal atrophy rate. ANTS software (www.picsl.upenn.edu/ANTS) was used to compute the symmetric deformation field (SyN method) between MRI_acq1 and MRI_acq2 using a normalised cross-correlation metric. The displacement of each voxel in MRI_acq1 was written in the WarpX, WarpY, and WarpZ images of the deformation fields. The parameters of our deformation-based morphometry included isotropic 2 mm (FWHM Gaussian kernel) image smoothing. Finally, for each vertex of the hippocampal surface (MRI_acq1), the dot product between the normal and the deformation field defined the signed displacement of the vertex (in mm) (Fig. 1B). The resulting values (divided by the duration between acquisition MRI_acq1 and MRI_acq2) represent the atrophy rate in mm/year. The texture was displayed on the 3D hippocampus in the radiological convention with visualisation software (anatomist, brainvisa.info/). The 3D mesh together with statistical maps is also available: brainsenses.x10host.com/hc.htm. The hippocampal volume was computed from the volumetric meshes. The technique described here was partly published in abstract form with preliminary results [22].

### Statistical Analyses

Statistical analyses were performed using the R software (www.cran.r-project.org). The permutation test was used to assess the significant atrophy rate within each group. Statistical maps are displayed on the 3D hippocampi as -log10(p-value) for above threshold. Group differences were assessed using the Wilcoxon signed-rank test, which does not require the normality of the data. For both type of tests, significance level (p<0.05) was corrected for multiple comparison (number of vertices) using Bonferroni correction.

### Hippocampal Subfields

**Figure 2:**
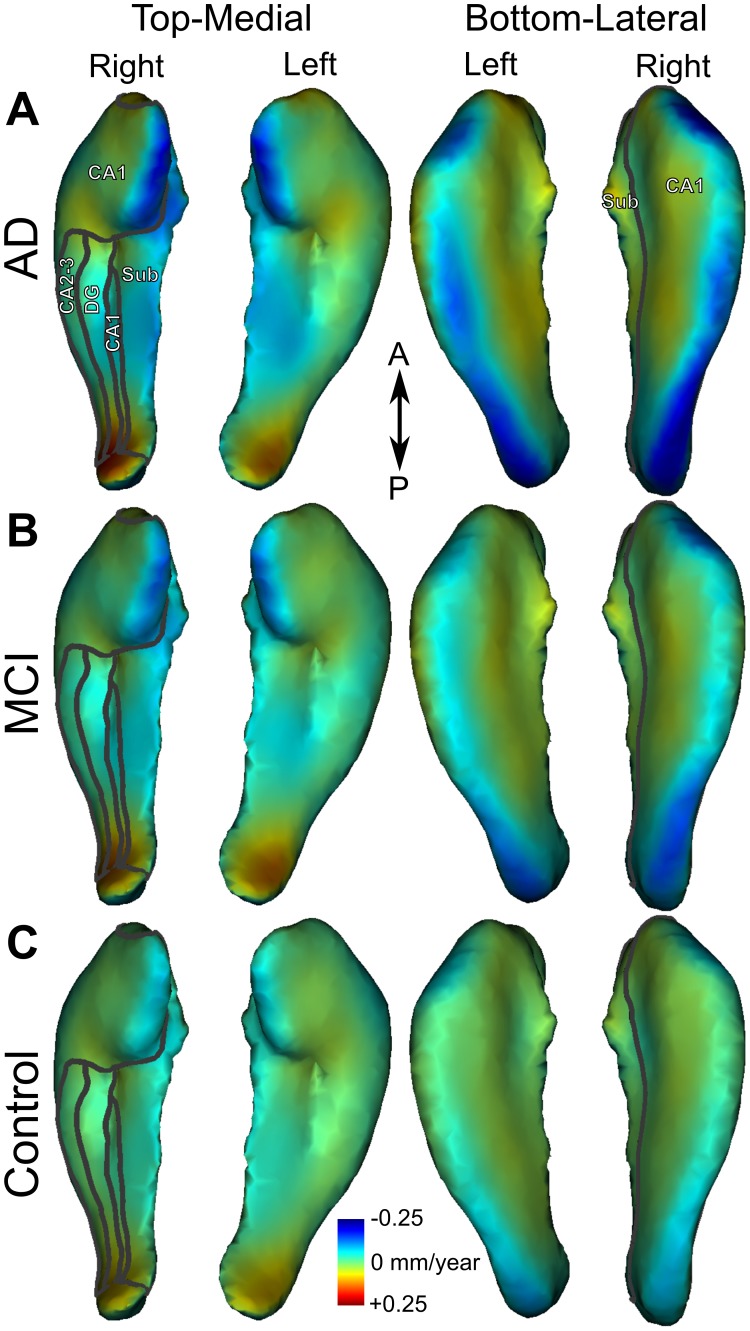
Mean atrophy rate. Mean atrophy rate in mm/year for the left and right hippocampi in patients with AD (**A**), MCI (**B**) and controls (**C**). On the right hippocampus, projected borders of subfields CA1, CA2–3, dentate gyrus (DG) and subiculum (Sub) are indicated with grey outlines.

Even though the hippocampal subfields are determined by histology and their boundaries are not visible on MRI, the subfields are often indicated on in vivo MRIs based solely on visual cues derived from histological images. However, a more reliable and unbiased subfield delineation can be achieved by using an independent MRI atlas. Hence, to help localise significant atrophy rates on the hippocampus, we projected the hippocampal subfields as defined from the high-resolution atlas of the human hippocampus [27] computed from post-mortem MRI at 9.4 Tesla (www.nitrc.org/projects/pennhippoatlas) onto our template using ICP algorithm. The borders of the subfields including CA1, CA2–3, the dentate gyrus, and the subiculum are illustrated as outlines on the 3D surface of the right hippocampus in Fig. 2A.

### Region of Interest Analysis

To further test for differences between pMCI and sMCI, we performed a Region of Interest (ROI) analysis of the atrophy rate. The ROIs were used to increase the sensitivity to distinguish patients with stable MCI from progressive ones. We defined the ROIs as clusters of vertices with significantly higher atrophy rates in AD than in control subjects; which make them independently defined from the MCI dataset. A total of four ROIs were defined both in the right (R1R-R4R) and left (R1L-R4L) hippocampi. R1 is the cluster of vertices in the medial side of the hippocampal head, R2 corresponds to the cluster in the medial side of the body, R3 represents the cluster in the lateral side of head, whereas R4 contains the clusters along the lateral side of the hippocampal body and tail. The ROIs therefore do not correspond to a specific hippocampal subfield derived from the high-resolution atlas (see section Hippocampal subfields).

Within each region, we computed the average atrophy rate for each MCI patient. The stable and progressive MCI groups were compared statistically using Wilcoxon test (significance level was adjusted for the number of ROIs with Bonferroni correction). Receiver Operating Characteristic (ROC) analysis was then performed using the R package pROC version 1.4.4 [28].

## Results

### Hippocampal Volume Change

We first examined the difference in hippocampal volume between MRI_acq1 and MRI_acq2. Estimations of hippocampal volumes (in mm3) for MRI_acq1 and the volume change between MRI_acq1 and MRI_acq2 (in mm3/year) are listed in Table 2. We found significant hippocampal volume loss rates in each group (Table 2). However, the loss was greater in patients with AD than in controls in both hippocampi (right: W = 5390, p-value = 1.501e-06; left: W = 4690, p-value = 0.004927). The volume loss was significantly higher in patients with MCI than in controls in the right hemisphere but not the left (right: W = 5273, p-value = 0.005484; left: W = 4719, p-value = 0.149 n.s.). Similarly, the AD group showed significantly greater volume loss than the MCI group in the right but not in the left hippocampus (right: W = 5507, p-value = 0.008535; left: W = 5190, p-value = 0.05936 n.s.). No significant difference in volume change was found between progressive and stable MCI (right: W = 1025, p-value = 0.06436 n.s.; left: W = 996, p-value = 0.1048 n.s.). Collectively, the volumetric measurements revealed significant loss in all groups and a larger loss in the AD and the MCI groups than in controls, but they failed to show significant difference between pMCI and sMCI.

**Table 2:** Summary of hippocampal volumetry.

Group	Volume MRI_acq1 (R/L)	Volume change per year (R/L)	Statistics and p-value (R/L)
2*Control (n = 85)	2402 (476)/	−9 (30)/	V = 774, p-value = 1.975e-06/
	2059 (404)	−6 (22)	V = 992, p-value = 0.0001267
2*MCI (n = 102)	2181 (468)/	−20 (31)/	V = 815, p-value = 2.079e-09/
	1946 (431)	−10 (30)	V = 1360, p-value = 3.115e-05
2*AD (n = 90)	2129 (446)/	−28 (41)/	V = 479, p-value = 1.403e-10/
	1805 (397)	−14 (34)	V = 978, p-value = 8.489e-06

Average hippocampal volume in mm3 (SD) at the first scan, average volume loss in mm3/year between the two scans for right and left (R/L) hippocampi (SD), and the statistical tests on volume loss (Wilcoxon test) in each group.

### Mapping the Rate of Atrophy

**Figure 3:**
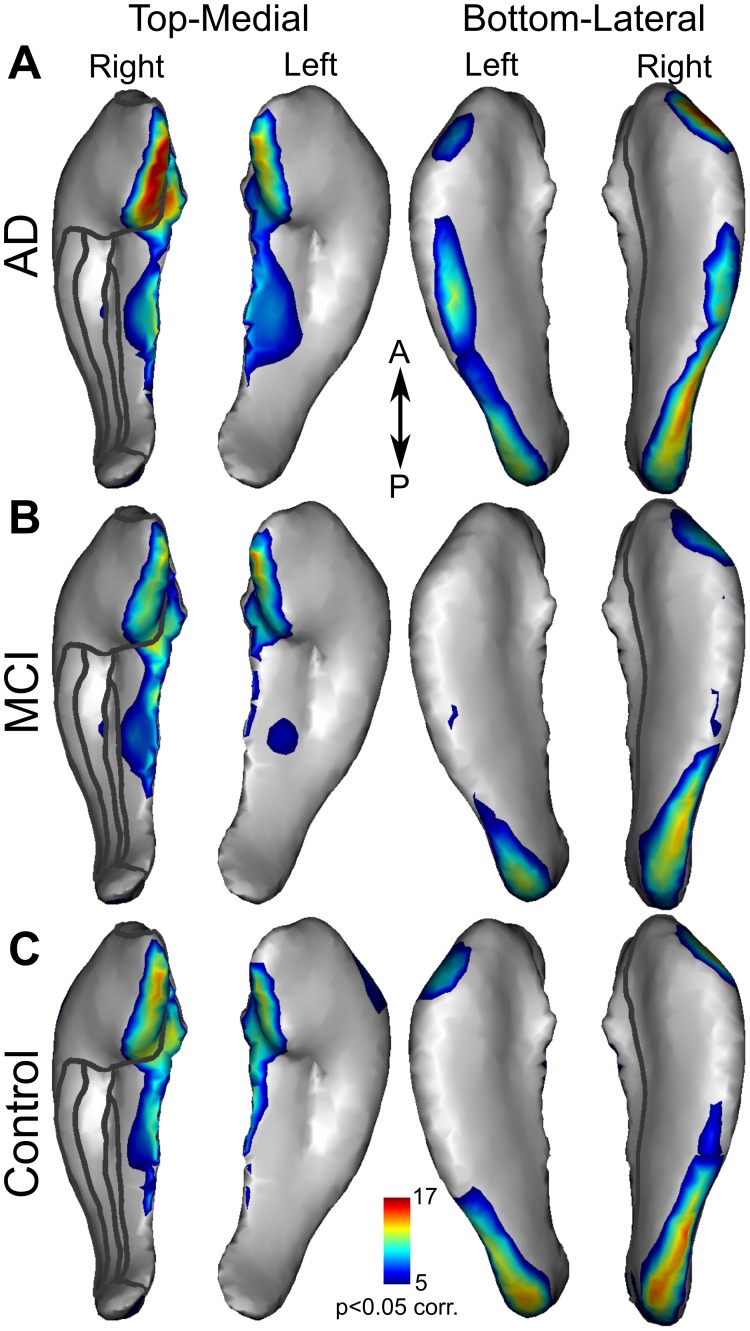
Significant atrophy rates. Statistical maps showing the -log(p-values) for significant (p
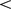
0.05 corr.) atrophy rates for the left and right hippocampi in patients with AD (**A**), MCI (**B**) and controls (**C**). Significance level (p
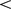
0.05 corr.) is indicated in the colour bar.

We computed averaged maps of hippocampal atrophy rates (mm/year) for patients with AD (Fig. 2A), MCI (Fig. 2B), and for controls (Fig. 2C). This mapping revealed a similar pattern of atrophy rates in all groups. Nonetheless, the rate of atrophy was the highest in the AD group (demonstrated by darker blue in Fig. 2), particularly in CA1 and subiculum. To assess atrophy rate significance, statistical maps were derived from a permutation test within each of the three populations (Fig. 3 A–C). Again, a similar pattern was found in the three groups. Significant atrophy occurred in the medial head and body and along the lateral side of the hippocampi. The highest significance was found in the medial head of the right hippocampus in the AD group (Fig. 3A and online 3D mesh, see Methods). Figures 2 and 3 suggest a similar pattern of atrophy rate among the three groups with different magnitudes that were further examined.

### Group Differences

**Figure 4:**
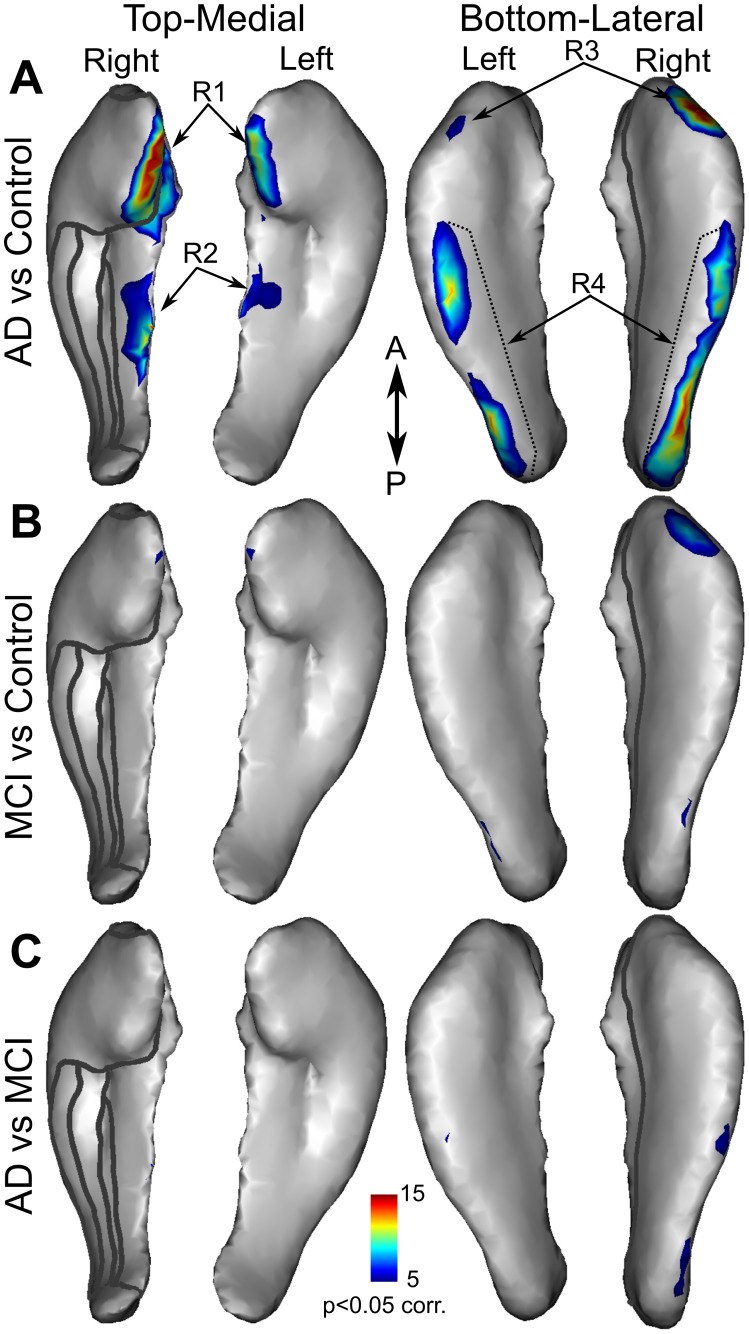
Statistical maps of atrophy rate differences among groups. Statistical maps showing significantly higher atrophy rate in AD versus control (**A**), MCI versus control (**B**), and AD versus MCI (**C**). Significance level (p
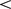
0.05 corr.) is indicated in the colour bar.

The significantly higher rate of atrophy in patients with AD than in controls (Fig. 4A and online 3D mesh, see Methods) was found mainly in the medial part of the head and body and along the lateral side of the hippocampi (Wilcoxon test, p
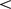
0.05 corr.). A higher atrophy rate in the MCI group as compared to controls (Fig. 4B) was found in much smaller regions, mainly on the lateral side of the hippocampal head. Finally, significant differences between the AD and MCI groups (Fig. 4C) were found in a few vertices along the lateral side.

### Stable versus Progressive Mild Cognitive Impairment

**Figure 5:**
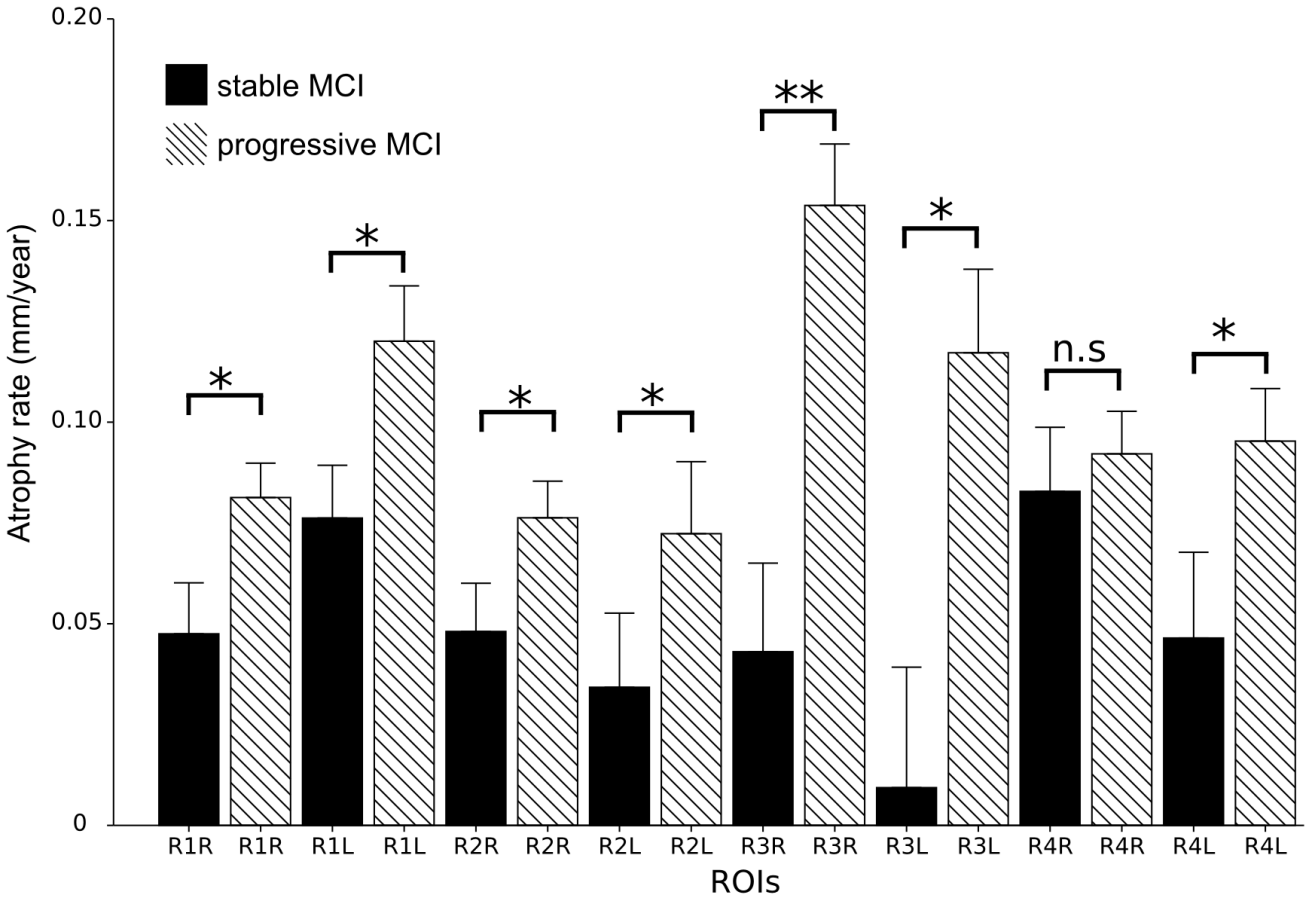
ROI analysis of atrophy rates in progressive and stable MCI. Bar plots of atrophy rates in the left and right hippocampal ROIs (shown in Figure 4) for pMCI (n = 39) and sMCI (n = 44). *: p
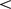
0.05, **: p
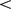
0.001 Bonferroni adjusted p-values.

The difference in atrophy rate between the pMCI and sMCI groups (Fig. 5) was only assessed within the regions showing significantly higher atrophy rate in AD compared with control in order to increase the sensitivity. These comparisons revealed significantly higher rates in pMCI in all but one ROI (Wilcoxon test, R1R: W = 518, p-value = 0.001951; R1L: W = 497, p-value = 0.001004; R2R: W = 563, p-value = 0.007208; R2L: W = 570, p-value = 0.00871; R3R: W = 453, p-value = 0.0002236; R3L: W = 519, p-value = 0.002011; R4R: W = 737, n.s; R4L: W = 543, p-value = 0.004110). The largest difference was found in the R3R ROI, which represents the antero-lateral part of the right hippocampus. Within this ROI, we performed an ROC analysis to determine how well the atrophy rate of this region is able to distinguish between pMCI and sMCI. The ROC analysis revealed 72.4% accuracy in discriminating the two groups, with 70.5% specificity and 74.4% sensitivity (area under the curve: 78.1%).

## Discussion

In the present study, we used two MRI scans to map the rate of hippocampal atrophy in patients with AD and MCI and in healthy elderly controls. We also measured the total hippocampal volume and compared its decrease among the three groups. We found the lowest baseline volume in the AD group, and the greatest in the group of healthy elderlies. The average hippocampal volume of patients with MCI was in between the other two groups. Looking at the volume loss over time, we found the fastest loss in the AD group. This was significantly higher than the loss in the normal group both in the left and the right hippocampi. The difference in volume loss between the AD and MCI groups was significant only in the right side. Similarly, only the right hippocampus showed greater volume loss in the MCI group than in controls. Comparison of the atrophy rates among the three subject groups revealed similar patterns. The regions with the highest rate of atrophy were located on the medial side of the hippocampal head and body and along the lateral side. These regions also showed significantly higher atrophy rates in patients with AD compared with controls. The difference in hippocampal atrophy rate between MCI and controls was located at the lateral side of the right hippocampal head, whereas the higher atrophy rate in AD compared to MCI was mainly found on the lateral side of the right hippocampal body and tail. To relate these regions to the histological subparts of the hippocampus, we used the atlas of the human hippocampus segmented from high-resolution MRIs [27]. Based on this atlas, the fastest atrophy occurred in the CA1 zone. Similarly, the strongest differences between the groups were localised in the CA1 zone and in the subiculum.

Our volumetric measurements are in accordance with previous studies measuring the hippocampal volume in different populations, namely sequential decrease in volume among healthy elderlies, MCI and AD patients [29]. We also found smaller volume in the left side compared to the right, as did many groups previously [30]–[32]. Our results are also in line with studies examining the volume loss over time [11], [31], [33]–[37]. They reported the greatest annual percent change in the hippocampal volume in patients with AD. This was significantly higher than that in patients with MCI and in normal subjects. Leung et al. [37] showed that the volume loss was even accelerating in the MCI group over time.

Although the difference in hippocampal volume had the same trend in many studies previously, the volume itself differed substantially. Groups, using the ADNI database of patients with AD and MCI and healthy elderly controls, reported hippocampal volumes in AD varying between 1600 [35] and 3000 mm3 [20]. In the present study, the hippocampal volume of AD patients was about 2000 mm3. The remarkable difference among studies might derive from the segmentation of the template hippocampus used to extract the structure in each individual. There are different recommendations for hippocampal segmentation which include different amount of the subiculum and the tail of the hippocampus [38]. It can also differ how far anterior the hippocampus is segmented inferior to the amygdala. Here we followed the recommendations of Franko et al. [23] and Insausti and Amaral [24]. These studies described landmarks visible on MRI which are based on the histological examination and post-mortem MRI of the same brains.

Recently, several studies have focused on 3D shape analyses and detailed hippocampal atrophy mapping, and compared these maps among healthy controls and patients with MCI and AD [20], [21], [32], [39]–[43]. Most of them used a single MRI scan and looked for differences in the hippocampal shape between healthy elderly and patients [20], [39], [40], [44]–[47], referring to this shape difference as atrophy. However, this requires the assumption that the patient group had the same hippocampal shape as the control group prior to the disease process, which might not be the case if the number of subjects is not sufficiently high. Importantly, more and more studies measure the atrophy using a longitudinal MRI dataset, allowing the visualisation of the hippocampal atrophy within each single subject over time [15], [19], [21], [32], [42]. This method does not require a template hippocampus computed by averaging of healthy subjects’ brains, therefore it is more robust for individual variability in the hippocampal shape. However, the methods could be biased by asymmetric deformation mapping [26], [48], which could explain the contradicting findings in the previous studies. We therefore examined the hippocampal atrophy performing an unbiased symmetric deformation field estimation.

One of the first studies comparing the hippocampal atrophy rate in patients with AD to that in healthy subjects was performed by Thompson et al. [19]. They reported similar atrophy rate patterns between the two groups but found significantly higher atrophy rates on the lateral side of the left hippocampal head in AD patients. We also found similar atrophy rate patterns among the groups; however, we found significantly higher rates in AD patients compared to controls not only on the lateral side of the head but also on the medial part of the head and body and the lateral side of the hippocampal body and tail. The hippocampal atrophy rate maps reported by other groups [21], [32], [42] differ more from our results. Morra et al. [21] mainly found atrophy on the superior and inferior parts of the body and on the tail of the hippocampus of AD patients, whereas in our study, the atrophy rate was higher on the lateral and medial sides relatively sparing the superior and inferior surfaces. However, when comparing the patients to healthy controls, the significantly different atrophy was located mainly on the inferior part of the head and on the lateral and medial sides of the hippocampus, which is closer to our findings. However, another study from the same group [20] reported significant difference between AD patients and controls on almost the entire surface of the hippocampus sparing only a line along the middle of the superior and inferior surfaces. Wang et al. [32], [39] compared patients with MCI (CDR = 0.5) to healthy controls. They found more inward deformation in patients on the superior side of the head and the inferior and lateral parts of the hippocampal body and tail. They also reported the inward deformation of these regions to predict conversion from healthy state to MCI [49]. Apostolova et al. [42] compared subjects who remained cognitively normal with those who converted to MCI or AD and showed significant differences in hippocampal atrophy rate in all parts of the hippocampus without subfield specificity (CA1–3 subfields and subiculum).

Although Gerardin et al. [40] used a single scan to examine the hippocampal shape, the topography of atrophy found in the present study is the most like their results. They found morphological differences between the hippocampi of patients with AD and those of controls, predominantly on the medial part of the head and the lateral part of the body and tail. However, the use of a single scan prevented the investigation of atrophy evolution. Our data suggest that the morphological differences among groups in their study may be the consequence of different atrophy rates. Costafreda et al. [43] also used a single scan to predict conversion from MCI to AD using a baseline atrophic phenotype. Similarly to our findings, the atrophy of the anterior part of the CA1 field best predicted the conversion, with 80% accuracy. We also found the lateral side of the hippocampal head (corresponding to CA1) to be the best predictor of the progression to AD from MCI based on the atrophy rate. Although the accuracy is slightly lower, our method can be used to identify patients at high risk of progression. This can help identify patients most likely to be helped by treatments that are mainly effective in the early phase of AD.

We found similar patterns of atrophy rate among the three subject groups which might suggest that certain hippocampal subregions are more prone to atrophy caused by normal or pathological ageing. However, the current method cannot distinguish between different sources of atrophy (underlying mechanisms of cell death). Therefore, we cannot speculate whether the similarity in atrophy pattern in AD compared to the controls would indicate that the disease simply accelerates the normal ageing. Braak and Braak [5] found that tau (neurofibrillary tangle and neuropil thread) deposition in the hippocampus first occurs on the medial and lateral side of the CA1 field in the early phase of AD (stage II, their Fig. 4); these regions are also more affected in the later phases than the middle portion of the CA (superior portion of the 3D hippocampus, see Methods) or the subiculum (infero-medial portion of the 3D hippocampus). Importantly, these regions that have the highest burden of neurofibrillary tangle and neuropil thread deposition correspond to those showing the highest atrophy rate in our study. This good correspondance between our results and the well-established neuropathology in AD supports a biological interpretation of the current mapping of atrophy rate.

Longitudinal studies are also necessary to evaluate atrophy progression at the individual level, which is the best way to objectively measure treatment effects. Jack and colleagues previously demonstrated that higher annual hippocampal volume loss correlated with worsening of the clinical status [11]. However, when comparing treated and untreated AD patients, Fox et al. [34] reported non-significant hippocampal volume change. Similarly, Wang et al. [41] did not find significant differences in hippocampal volume or surface deformation between treated and untreated MCI patients. Their method of averaging the deformation within each subfield assumes homogeneous atrophy. This might decreases the sensitivity to mild treatment effects. The present method is more sensitive to local deformation, which makes it more appropriate for use in clinical trials.

Beside its high sensitivity to local deformation, our method is also free of biases arising from asymmetric global normalization [26]. In a very recent study, Lorenzi and colleagues [48] developed another local symmetric registration algorithm robust to intensity bias. Their method will be useful for future longitudinal studies. Even though we used a semi-automated segmentation of the hippocampus at baseline, this step could be replaced with a fully-automated method. Several groups have developed fully-automated hippocampal segmentation tools [50]–[52] and some are recently available using FIRST/FSL and FreeSurfer softwares. Although our method is more time-consuming for large cohorts of patients and requires anatomical knowledge, it enabled us to use hippocampal boundaries as described by Insausti and Amaral [24].

In conclusion, our MRI-based regional hippocampal atrophy maps are in high agreement with the known AD histopathology. This shows that longitudinal MRI and particularly the measurement of local hippocampal atrophy rate are reliable methods to investigate AD. Additionally, we describe an unbiased method to objectively evaluate AD progression that can be used in clinical trials to test novel disease-modifying drugs and measure their efficacy.
